# Enhanced Liver Fibrosis Score for Diagnosing Liver Fibrosis in Chronic Hepatitis

**DOI:** 10.3390/diagnostics14131317

**Published:** 2024-06-21

**Authors:** Nobuharu Tamaki, Kenta Takaura, Mayu Higuchi, Yutaka Yasui, Jun Itakura, Kaoru Tsuchiya, Hiroyuki Nakanishi, Namiki Izumi, Masayuki Kurosaki

**Affiliations:** Department of Gastroenterology and Hepatology, Musashino Red Cross Hospital, Tokyo 180-8610, Japan

**Keywords:** enhanced liver fibrosis (ELF) score, chronic hepatitis, liver biopsy, FIB-4

## Abstract

**Background and aims:** The enhanced liver fibrosis (ELF) score is a blood test that combines three markers linked to liver fibrosis. The utility of the ELF score has been demonstrated primarily in Western countries, but whether it is useful in areas with a high number of elderly people suffering from chronic liver disease has yet to be determined. **Methods:** This is a prospective study that included 373 consecutive patients who underwent a liver biopsy and had their ELF score measured on the same day. The diagnostic accuracy of the ELF score for liver fibrosis and the effect of age on the ELF score were investigated. **Results:** The median (interquartile) ELF scores in F0, F1, F2, F3, and F4 are 8.7 (8.2–9.2), 9.3 (8.8–10.0), 10.1 (9.4–10.7), 10.7 (9.9–11.2), and 12.0 (11.2–12.7), respectively. ELF scores increased with increasing liver fibrosis stage (*p* < 0.001). The diagnostic accuracy of the ELF score and FIB-4 for significant fibrosis (F2–4) and advanced fibrosis (F3–4) was comparable, but the ELF score had a higher diagnostic accuracy for cirrhosis (F4) than FIB-4. When patients were stratified by age of 60 years, the median ELF score did not differ by age in F2, F3, and F4. However, the median FIB-4 increased in patients with ≥60 years compared to those with <60 years in all fibrosis stages. **Conclusions**: ELF score has high diagnostic accuracy for liver fibrosis, regardless of age, and it could be used as a primary screening method.

## 1. Introduction

Chronic liver disease, such as chronic hepatitis C, chronic hepatitis B, and steatotic liver disease, progresses to hepatocellular carcinoma (HCC) and liver decompensation, with HCC being one of the leading causes of cancer-related death worldwide [1,2]. Chronic liver disease and its complications have emerged as an economic and health burden [3,4]. Therefore, it is critical in clinical practice to identify patients at high risk of HCC and decompensation and initiate treatment.

Liver fibrosis is the most important risk factor for HCC development or decompensation, so an accurate assessment of liver fibrosis status in patients with chronic hepatitis is critical [5,6,7]. Although liver biopsy is the gold standard for evaluating liver fibrosis, it has several drawbacks, including invasiveness and cost [8]. Furthermore, chronic liver disease is common in the general population, and liver biopsy is difficult to perform in large groups. To address this limitation, noninvasive methods for diagnosing liver fibrosis and pathological features have developed and are now widely used in clinical practice [9,10]. As noninvasive methods, blood test-based methods and imaging-based methods have emerged and blood test-based methods are suitable as a first-step screening [11,12]. The enhanced liver fibrosis (ELF) score is a blood test that combines tissue inhibitor of metalloproteinases 1 (TIMP-1), amino-terminal propeptide of type III procollagen (PIIINP), and hyaluronic acid (HA) [13,14]. Several studies have demonstrated the usefulness of the ELF score in diagnosing liver fibrosis, and it has been reimbursed in Japan since February 2024 [15,16,17,18]. However, there are many elderly patients with chronic liver disease in Japan, and it is unclear whether ELF can be used to diagnose liver fibrosis in these patients. To address a current gap in knowledge, this study investigated the diagnostic ability of the ELF score for liver fibrosis, with a particular emphasis on age.

## 2. Methods

### 2.1. Study Protocol

This is a prospective study that was conducted from October 2012 to September 2015 at Musashino Red Cross Hospital. The study included consecutive patients with chronic liver disease who underwent liver biopsy and had their ELF scores measured on the same day. A total of 405 patients were included in the study. Among them, 32 patients with poor liver biopsy specimens for diagnosing liver fibrosis stage were excluded, leaving 373 patients who underwent liver biopsy and had their ELF score measured.

All patients provided written informed consent. The study methods followed the ethical guidelines of the Declaration of Helsinki, and the study was approved by the ethical committee of Musashino Red Cross Hospital (approved number: 23021, approval date: 25 January 2012).

### 2.2. Liver Biopsy Assessment

Liver biopsy specimens were obtained through laparoscopic or percutaneous ultrasound-guided liver biopsy with 13G or 15G needles. The specimens were fixed, paraffin-embedded, and stained with hematoxylin–eosin. The median (interquartile range [IQR]) biopsy specimen length was 15 (12–18) mm. All liver biopsy samples were independently evaluated by two senior pathologists who were unaware of the clinical data. Liver fibrosis stage was classified using the METAVIR score as F0: no fibrosis, F1: portal fibrosis but no septa, F2: portal fibrosis and few septa, F3: multiple septa but no cirrhosis (bridging fibrosis), and F4: cirrhosis, F2–4: significant fibrosis, F3–4: advanced fibrosis, F4: cirrhosis.

### 2.3. ELF Score and FIB-4

Blood samples were collected on the same day as the liver biopsy or the day before. The ELF score is computed using the following formula: The ELF score = 2.278 + 0.851 × ln(HA) + 0.751 × ln(PIIINP) + 0.394 × ln(TIMP-1). Furthermore, the FIB-4 index is another noninvasive marker for liver fibrosis, and its usefulness has been documented [19,20]. We also computed FIB-4 with the following formula: The FIB-4 index = (age × AST)/(platelet count [109/L] × (ALT)1/2), where AST is aspartate aminotransferase and ALT is alanine aminotransferase.

### 2.4. Statistical Analyses

Differences in ELF scores per stage and by age were compared using the Mann–Whitney U test. Receiver operating characteristic (ROC) curves were created, and the area under the ROC curve (AUROC) was computed. The AUROC of ELF score and FIB-4 was compared using the DeLong test. Statistical significance was defined as *p*-values of <0.05. All statistical analyses were performed using EZR (Saitama Medical Center, Jichi Medical University, Shimotsuke, Japan), a graphical user interface for R version 3.2.2 (The R Foundation for Statistical Computing, Vienna, Austria).

## 3. Results

### 3.1. Patient Characteristics

The study included 373 patients with chronic hepatitis who underwent a liver biopsy and had their ELF scores measured. The patient characteristics are shown in Table 1.

The median (IQR) age was 60 (48–66) years, and 47% of patients were male. The rates of chronic hepatitis C, chronic hepatitis B, and non-viral hepatitis were 55.8%, 22.5%, and 21.7%, respectively. Regarding liver fibrosis stage determined by liver biopsy, fibrosis stages 0, 1, 2, 3, and 4 were 36 (9.7%), 138 (37.0%), 86 (23.1%), 85 (22.7%), and 28 (7.5%), respectively. The median (IQR) ELF and FIB-4 scores were 9.9 (9.0–10.7) and 20.9 (1.35–3.49), respectively.

### 3.2. ELF Score and Fibrosis Stage

**Figure 1:**
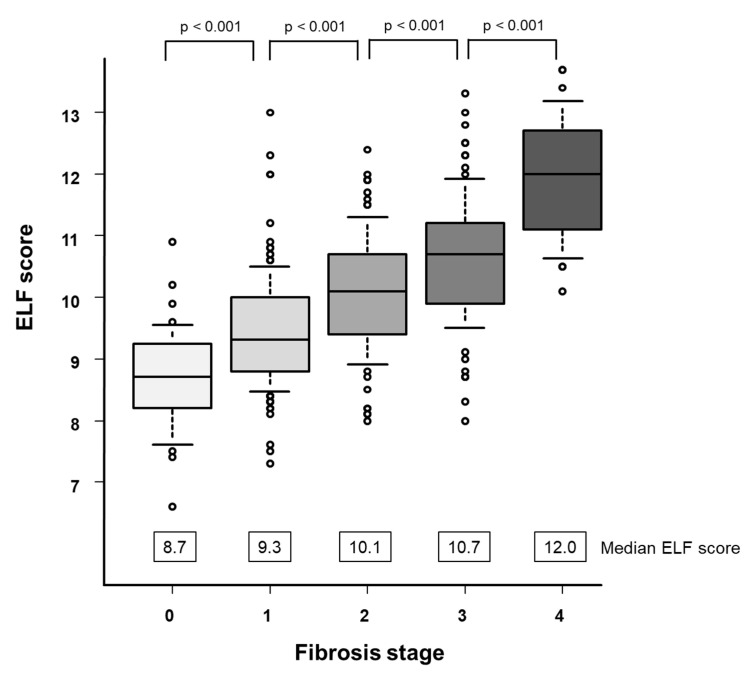
Correlation between the ELF score and fibrosis stage. The box plots of the ELF score are shown according to each fibrosis stage. The bottom and top of each box represent the 25th and 75th percentiles, giving the interquartile range. The line through the box indicates the median value and the error bars indicate the 10th and 90th percentiles.

ELF scores for each fibrosis stage are shown in Figure 1.

The median (IQR) ELF scores in F0, F1, F2, F3, and F4 are 8.7 (8.2–9.2), 9.3 (8.8–10.0), 10.1 (9.4–10.7), 10.7 (9.9–11.2), and 12.0 (11.2–12.7), respectively (Figure 1). ELF scores increased as the liver fibrosis stage progressed (*p* < 0.001).

### 3.3. Comparison Diagnostic Accuracy between ELF Score and FIB-4

**Figure 2:**
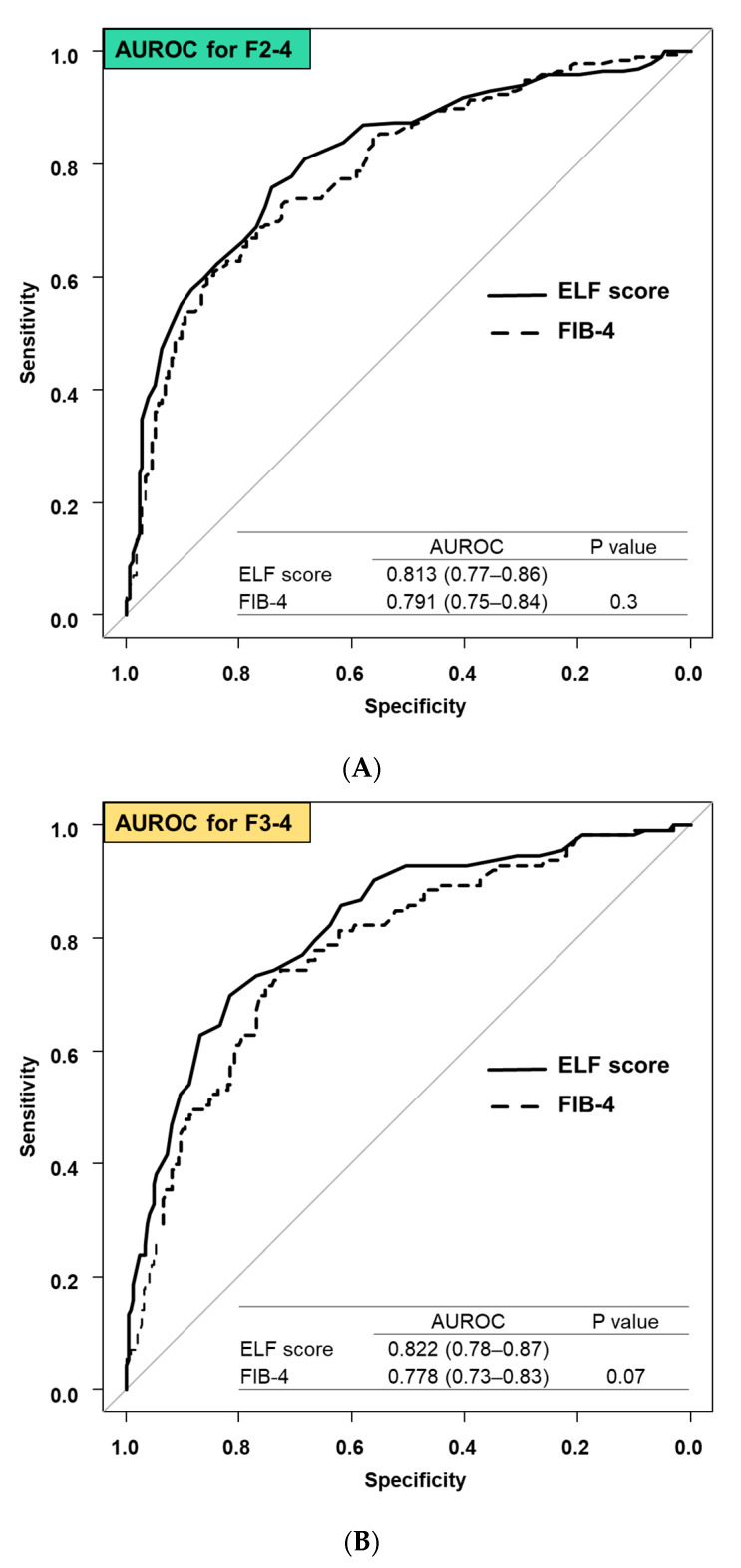

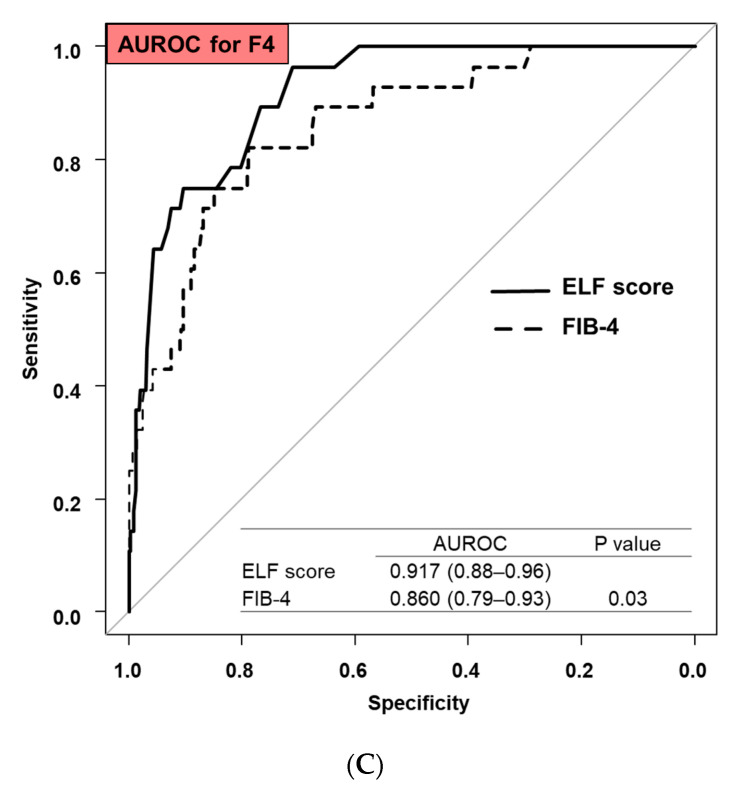
Comparison of AUROC for diagnosis of liver fibrosis between the ELF score and FIB-4: (**A**) diagnosis of significant fibrosis (F2–4), (**B**) diagnosis of advanced fibrosis (F3–4), and (**C**) diagnosis of cirrhosis (F4).

The diagnostic accuracy of ELF score and FIB-4 for significant fibrosis (F2–4), advanced fibrosis (F3–4), and cirrhosis (F4) were compared. The AUROCs (95% confidence interval [CI]) for ELF score and FIB-4 for significant fibrosis were 0.813 (0.77–0.86) and 0.791 (0.75–0.84), respectively (*p* = 0.3, Figure 2A).

Similarly, the AUROCs (95% CI) of ELF score and FIB-4 for advanced fibrosis were 0.822 (0.78–0.87) and 0.778 (0.73–0.83), respectively (*p* = 0.07, Figure 2B), and for cirrhosis were 0.917 (0.88–0.96) and 0.860 (0.79–0.93), respectively (*p* = 0.03, Figure 2C).

The diagnostic accuracy of the ELF score and the FIB-4 for significant and advanced fibrosis was equivalent, but the ELF score was more accurate for cirrhosis than the FIB-4. In a subgroup analysis, the diagnostic accuracy for cirrhosis in patients with chronic hepatitis B, chronic hepatitis C, and non-viral hepatitis was investigated. The AUROCs (95% CI) of ELF score and FIB-4 for cirrhosis were 0.932 (0.94–1) and 0.776 (0.39–1) in patients with chronic hepatitis B (*p* = 0.3), 0.906 (0.85–0.96) and 0.861 (0.49–0.94) in patients with chronic hepatitis C (*p* = 0.1), and 0.939 (0.86–1) and 0.837 (0.62–1) in patients with non-viral hepatitis (*p* = 0.2), respectively.

### 3.4. Effect of Age on ELF Score and FIB-4

**Figure 3:**
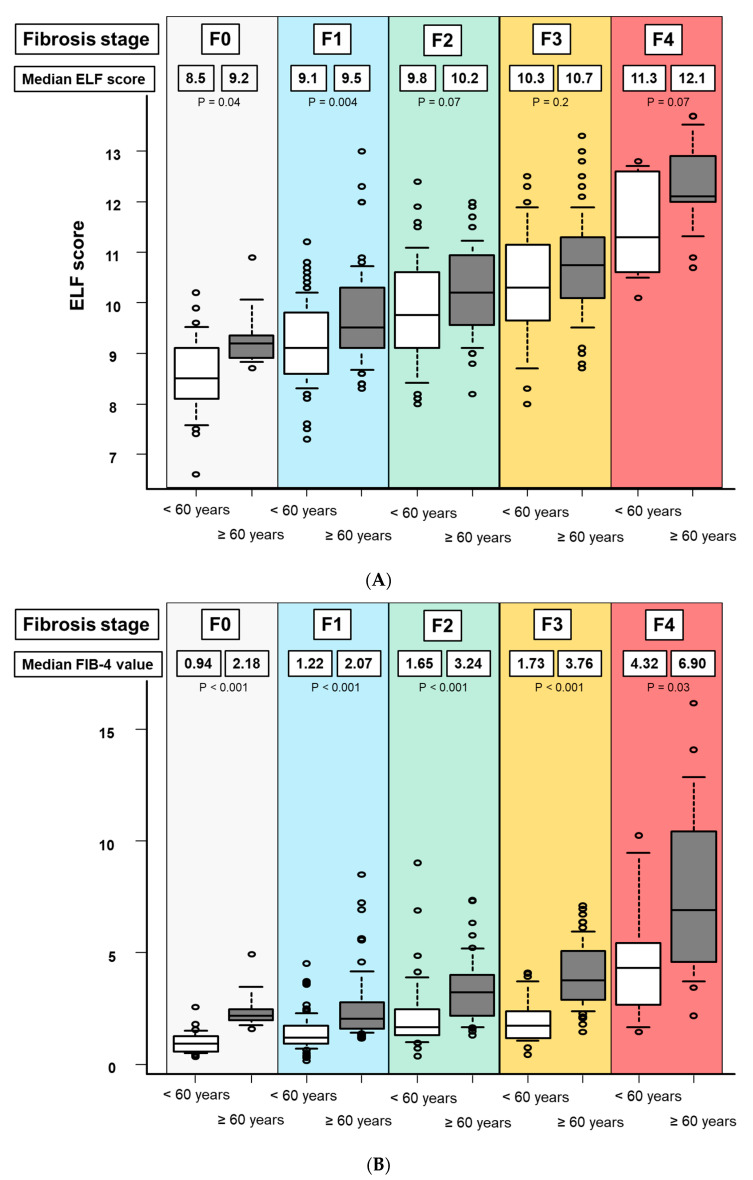
Effect of age on the ELF score and FIB-4. Patients are stratified by age of 60 years and fibrosis stage. (**A**) effect of age on the ELF score and (**B**) the effect of age on FIB-4. The bottom and top of each box represent the 25th and 75th percentiles, giving the interquartile range. The line through the box indicates the median value and the error bars indicate the 10th and 90th percentiles.

Patients were stratified by age (60 years was the median age of the study cohort), and the effect of age on ELF score and FIB-4 was studied. In patients with F0, the median ELF score (IQR) in patients aged < 60 years and ≥60 years were 8.5 (8.1–9.1) and 9.2 (8.9–9.3), respectively (*p* = 0.04, Figure 3A).

Similarly, in patients with F1, F2, F3, and F4, the median ELF score (IQR) in patients with age < 60 years and ≥60 years were 9.1 (8.6–9.7) and 9.5 (9.1–10.2) for F1 (*p* = 0.004), 9.8 (9.1–10.6) and 10.2 (9.6–10.9) for F2 (*p* = 0.07), 10.3 (9.6–10.9) and 10.7 (10.1–11.2) for F3 (*p* = 0.2), and 11.3 (10.6–12.6) and 12.1 (12.0–12.9) for F4 (*p* = 0.07), respectively. In patients with F2, F3, and F4, the ELF score was unaffected by age. Regarding FIB-4, in patients with F0, F1, F2, F3, and F4, the median FIB-4 (IQR) in patients with age <60 years and ≥60 years were 0.94 (0.57–1.29) and 2.18 (1.98–2.47) for F0 (*p* < 0.001), 1.22 (0.94–1.74) and 2.07 (1.60–2.80) for F1 (*p* < 0.001), 1.65 (1.31–2.44) and 3.24 (2.19–3.88) for F2 (*p* < 0.001), 1.73 (1.18–2.38) and 3.76 (2.87–5.07) for F3 (*p* < 0.001), and 4.32 (2.69–5.43) and 6.90 (4.60–10.4) for F4 (*p* = 0.03), respectively (Figure 3B).

The median FIB-4 increased in patients with ≥60 years than those with <60 years in all fibrosis stages.

## 4. Discussions

### 4.1. Main Findings

In this prospective study, we found that the ELF score has high diagnostic accuracy for liver fibrosis in patients with chronic hepatitis. Furthermore, because the ELF score can be used regardless of age, it could be used as a first screening method.

### 4.2. In Context with Published Literature

The utility of the ELF score has been reported in numerous studies, primarily in Western countries, and the ELF score is recommended as a screening modality for liver fibrosis in Western guidelines [21,22], while in Japan, there are few studies investigating the utility of ELF scores, more validation studies in the Japanese population are required [23,24,25]. In this study, we demonstrated the high diagnostic accuracy of the ELF score for all fibrosis stages, thereby strengthening the utility of the ELF score in the Japanese population. A Japanese guideline recommends FIB-4 as the first-line screening modality for liver fibrosis, and it is used in clinical practice [26]. Therefore, we compared the diagnostic accuracy of liver fibrosis between the ELF score and the FIB-4 in this study. The diagnostic accuracy for significant and advanced fibrosis was similar between the two methods (Figure 2A,B), and the diagnostic accuracy of the ELF score for cirrhosis was significantly higher than that of FIB-4 (Figure 2C). Therefore, the findings support the use of the ELF score to diagnose liver fibrosis.

FIB-4 is widely used in clinical practice, but it is well understood that FIB-4 values are strongly influenced by age, and age-specific thresholds of FIB-4 have been proposed [27,28]. Furthermore, because Japan has a large number of elderly patients with chronic liver disease, FIB-4 as a first screening method may be difficult to implement in such an aging society. Therefore, age-independent diagnostic methods for liver fibrosis are becoming increasingly important, particularly in Japan. The effect of age on ELF has not been fully evaluated in previous research and was therefore examined in this study [29]. FIB-4 was significantly higher in all fibrosis stages in patients with ≥60 years, suggesting the need for an age-specific threshold, as previously reported [27]. Conversely, ELF scores showed no difference by age in patients with F2, F3, and F4. Therefore, ELF scores may be more useful in Japan, which has a large elderly population.

In this study, some patients with poor liver biopsy specimens for diagnosing the liver fibrosis stage were excluded. Liver biopsy has several limitations including sampling error or inter- and intra-observer variability. As liver fibrosis assessment by blood tests such as ELF score is more objective than liver biopsy and is easy to assess, it is more valid, especially in primary care. Although we demonstrate the usefulness of the ELF score for the diagnosis of liver fibrosis, long-term follow-up is needed in a future study to investigate the association between ELF score and the development of complications, including decompensation or HCC [24,25].

### 4.3. Strengths and Limitations

This is a prospective study in which consecutive patients with chronic hepatitis were enrolled. A further strength of the study was that liver fibrosis is assessed using a liver biopsy in all cases and ELF scores are measured on the same day as the liver biopsy. As a limitation, patients with chronic hepatitis from multiple etiologies are included. Because the number of patients was not sufficient to evaluate each etiology and the study was conducted in a single center, a multicenter study with increasing numbers of patients is needed to determine the accuracy of each etiology.

### 4.4. Future Implications

In this study, we demonstrated that the ELF score has high diagnostic accuracy for each fibrosis stage, regardless of age. Identifying patients at high risk of advanced fibrosis and cirrhosis among a large number of chronic hepatitis patients remains an unmet need. Initial screening in primary care is critical for identifying high-risk cases among a large population of chronic hepatitis patients [30]. Because a blood test can be easily measured without the use of a specific modality, blood tests are useful for initial screening. Based on the findings of this study, the ELF score can be used as a first screening method. The ELF score may be more useful than the widely used FIB-4, particularly in Japan, which has a large elderly population.

To summarize, the ELF score has high diagnostic accuracy for liver fibrosis, regardless of age, and it could be used as a first screening method.

## Figures and Tables

**Table 1:** Patient characteristics.

		*n* = 373
Age, years		60 (48–66)
Males		175 (47%)
Etiology	HCV	208 (55.8%)
	HBV	84 (22.5%)
	Non-viral	81 (21.7%)
Fibrosis stage	0	36 (9.7%)
	1	138 (37.0%)
	2	86 (23.1%)
	3	85 (22.7%)
	4	28 (7.5%)
Albumin, g/dL		4.2 (3.9–4.4)
AST, IU/L		43 (31–68)
ALT, IU/L		49 (31–86)
Platelet counts, 10^9^/L		175 (135–214)
FIB-4		2.09 (1.35–3.49)
ELF score		9.9 (9.0–10.7)
Continuous values are shown in median (interquartile range).		

HCV, hepatitis C virus; HBV, hepatitis B virus; AST, aspartate aminotransferase; ALT, alanine aminotransferase; ELF, enhanced liver fibrosis.

## Data Availability

The data presented in this study are available on request from the corresponding author, upon reasonable request.
